# The effect of latitudinal gradient on the species diversity of Chinese litter-dwelling thrips

**DOI:** 10.3897/zookeys.417.7895

**Published:** 2014-06-18

**Authors:** Jun Wang, Xiaoli Tong, Donghui Wu

**Affiliations:** 1 Key Laboratory of Wetland Ecology and Environment, Northeast Institute of Geography and Agroecology, Chinese Academy of Sciences, Changchun 130102, China; 2 Department of Entomology, South China Agricultural University, Guangzhou, China; 3 College of Plant Science, Jilin University, Changchun 130062, China

**Keywords:** Soil invertebrate, leaf-litter thrips, species diversity, latitudinal gradient, global distribution pattern, China

## Abstract

To understand the global distribution patterns of litter-dwelling thrips, a total 150 leaf litter samples were collected from 6 natural reserves located in three climatic regions, temperate, subtropical and tropical. The results showed the relative abundance of Thysanoptera was over 3.0% in 4 natural reserves from subtropical and tropical zone, and reached 5.9% in one tropical reserve, only less than Acarina and Collembola. In contrast it was only 0.3% in the warm temperate natural reserves, and no thrips were collected in a mid temperate reserve. The order on the average species numbers per plot of litter thrips was tropic > subtropics > temperate (n=25, *p*<0.05). Mean density of litter thrips per plots in the tropics and subtropics was significantly higher than that in the temperate region (n=25, *p*<0.05), but the average density was not significantly different between tropical and subtropical zones (n=25, *p*>0.05). The diversity of litter thrips in the tropics and subtropics was much higher than that in the temperate area based on comparsions of Shannon-Wiener diversity index (*H*’), Pielou eveness index (*J*), and Simpson dominance index (*D*). All of these results indicated that litter-dwelling thrips lived mainly in tropical and subtropical regions; meanwhile, species number and relative abundance increased with decreasing latitude.

## Introduction

Global distribution patterns of organisms have become a hot research topic in recent years due to increasing concerns about the global loss of species richness (Gaston 2000). The cogent statement of the increase in species diversity from polar to equatorial regions was confirmed by plants and vertebrate animals (Willig et al. 2003), but little is known about global pattern in species level of soil invertebrates, although these are recognized as one of the most species rich groups on earth (Wardle et al. 2004; Fierer et al. 2009). Maraun et al. (2007) investigated the latitudinal diversity gradient in a soil taxon, oribatid mites, and made a negative conclusion that diversity increases from the boreal to the warm temperate regions, but did not increase further in the tropics. Recently, Wu et al. (2011) used a molecular approach to analyze samples from 11 locations worldwide and suggested that there may be an inverse relationship between above-ground plant biodiversity and soil invertebrate animal biodiversity.

Litter-dwelling thrips is a group of thysanopteran insects that have adopted the habitat of forest litter or surface soil where they feed only on either fungal hyphae or fungal spores during the early stages of leaf decay (Mound 2005; Wang and Tong 2012). The diversity of these litter thrips is usually related to environmental factors, including temperature and humidity of the soil, the plant species that produce the litter (Tree and Walter 2012) and the species of fungi involved in decomposition (Ananthakrishnan 1996). This diversity is a potential indicator to assess changes in the forest environment (Mound 1977). A majority of litter–dwelling thrips species are wingless, usually have weak migratory ability, and can well provide materials for analyzing the fauna and zoogeography (Mound 1970). Another important role was played by some species which could be effective in the natural control particularly those efficient spore feeders of plant pathogenic fungi (Ananthakrishnan 1981).

Most publications of litter-dwelling thrips species were descriptions of new genera and species, sometimes with little information on their vegetation or microhabitat associations (Dang et al. 2013; Haga 1973; Mound 1970, 1976, 1977, 2002, 2013; Mound and Palmer 1983; Mound et al. 2013; Okajima 1981, 1994, 1998; Okajima and Urushihara 1992; Wang and Tong 2011; Wang et al. 2007, 2013). Mound (2002) indicated that litter–dwelling thrips are particularly diverse in the subtropics and tropics, with up to 50% of thysanopteran species in these regions. For example, almost 50 species of litter thrips were described from a single area of forest 50 km in diameter in southern Brazil (Mound 1977). Few quantitative field studies have been published on litter thrips. The only published quantitative field studies of litter thrips were conducted in subtropical China (Wang et al. 2008; Wang and Tong 2012) and in Australia (Tree and Walter 2012). In China, Wang and Tong (2012) found Thysanoptera constituted more than 1% of total litter–dwelling macroinvertebrate individuals extracted with modified Tullgren funnels. Species richness and abundance of litter-dwelling thrips gradually increased from July to December, and then declined rapidly. Litter-dwelling thrips were found only in the litter layer and upper soil layer (0-5 cm in depth) and were entirely absent in deeper soil layers. Tree and Walter (2012) found leaf-litter thrips were much more common and diverse in dry sclerophyll forest than in wetter forest types in subtropical southeast Queensland, Australia. The species diversity in soil fauna has been studied in temperate regions for more than 50 years, but with scarcely any mention of thrips (Wallwork 1976; Hattenschwiler et al. 2005). This lack of reference to thrips raises the question whether or not litter-dwelling thrips are distributed only in tropical and sub tropical regions.

To determine whether or not species diversity of litter-dwelling thrips alters at higher latitudes, we collected litter samples from six natural reserves which are located respectively in the temperate, subtropical and tropical zones, along a 4100 km latitudinal gradient in East China. The observations are also interesting from the point of view of understanding geographical scale differences in ecology, and responses of ecosystems to global warming.

## Material and methods

### Locations

**Figure 1.**
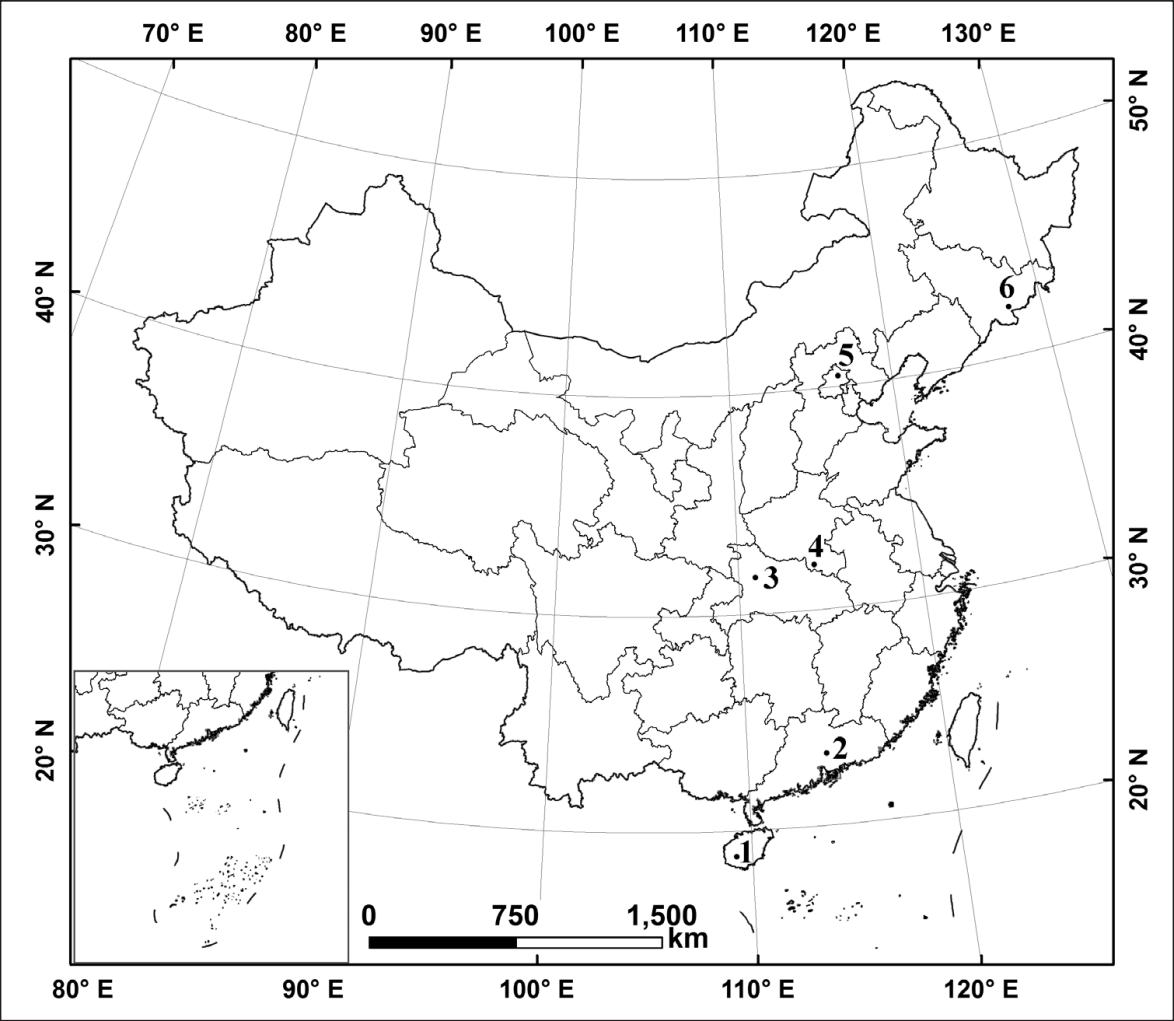
Locations of sampling stations in China. **1** Wuzhishan Natural Reserve, Hainan Province **2** Nankunshan Natural Reserve, Guangdong Province **3** Shennongjia Natural Reserve, Hubei Province **4** Jigongshan Natural Reserve, Henan Province **5** Yunmengshan National Forest Park, Beijing **6** Changbaishan Natural Reserve, Jilin Province.

This study was conducted on six different natural reserves along a broad latitudinal gradient China (Figure 1): Wuzhishan Natural Reserve (Hainan Province), Nankunshan Natural Reserve (Guangdong Province), Shennongjia Natural Reserve (Hubei Province), Jigongshan Natural Reserve (Henan Province), Yunmengshan National Forest Park (Beijing), Changbaishan Natural Reserve (Jilin Province). Natural conditions are summarized in Table 1.

**Table 1.** Natural conditions in the locations of sampling stations.

	1*	2	3	4	5	6
Latitude (N)	18°51'	23°38'	31°25'	31°50'	40°33'	41°55'
Longitude (E)	109°42'	113°50'	110°20'	114°05'	116°40'	127°40'
Annual average temperature (°C)	23	21	14	15	9	3
Annual average precipitation (mm)	2400	2163	1750	1119	700	680
Type of forest	Tropical rain forest	Evergreen seasonal rain forest	Deciduous broad-leaf forest	Deciduous broad-leaf forest	Deciduous broad-leaf forest	Pine and broad-leaf mixed forest
Zone of temperature	Tropic	Subtropics	Subtropics	Subtropics	Temperate	Temperate
Biogeographic region	Oriental region	Oriental region	Oriental region	Palearctic region	Palearctic region	Palearctic region

*1. Wuzhishan Natural Reserve, Hainan Province; 2. Nankunshan Natural Reserve, Guangdong Province; 3. Shennongjia Natural Reserve, Hubei Province; 4. Jigongshan Natural Reserve, Henan Province; 5. Yunmengshan National Forest Park, Beijing; 6. Changbaishan Natural Reserve, Jilin Province.

### Sampling

Wang et al. (2012) suggested that species diversity of litter-dwelling thrips was highest in October and December. Species diversity of litter thrips and other litter macro-invertebrates was quantitatively sampled from six natural reserves in October from 2007 to 2010. In each sampling, 5 plots (10 × 10 m) were randomly selected, and the distance between each sample plot was more than 50 m; 5 quadrate litter samples (50 × 50 cm) were selected in each plot – four in each corner and one in the center. A total of 150 litter samples were then placed in labeled plastic bags and extracted in the laboratory by means of a modified Tullgren funnel.

### Sorting and identification

Litter-dwelling thrips and other soil macro-invertebrates were extracted with the modified Tullgren funnels, using 60 W bulbs suspended 10 cm above the top of the samples over 10 hrs until the litter dried and became fragile. Specimens were then preserved in 75% ethanol. All extractions were completed within 3 days. The macro-invertebrate samples were sorted to Order level, and counted under a dissecting microscope. The adults of leaf-litter thrips were identified to species, and the larval stages of thrips were separated under the category of “thrips larvae” and counted separately.

### Data analysis

Species richness, density (individuals/m^2^), relative abundance and frequency were applied to indicate the diversity of litter-dwelling thrips. Relative abundance refers to the total number of specimens for a particular species divided by the total number of all litter thrips, while frequency expresses the number of individuals a species collected in a month, divided by the total number of months. A “dominant group” is defined as having a relative abundance of more than 10%; the relative abundance of “ordinary groups” is between 1% and 10%; “rare group” is less than 1%. The density of each thrips species in each month was the mean of 25 quadrate samples from 5 plots and presented as Mean ± standard errors.

Shannon-Wiener diversity index (*H*’) = -∑(*N_i_* / *N*) ln (*N_i_* / *N*)

Simpson dominance index (D) = ∑(*N_i_* / *N*)^2^

Pielou evenness index (*J*) = *H*’ / ln *S*

In the above, *N_i_* is the number of individuals of species *i*; *N* is the total number of individuals of all the species; *S* is the number of species (Wang et al. 2008). The analyses were carried out using SPSS ver 12.0.

## Results

### Species composition and relative abundance of litter-dwelling thrips

A total of 53,353 individuals of litter-dwelling invertebrate were collected in 150 samples, belonging to 30 groups in 10 classes under 3 phyla. Acarina and Collembola accounted for more than 10.0% of the total individuals of litter invertebrates and were considered to be “the dominant groups”. Relative abundance of litter-dwelling thrips was distinctly different in the different natural reserves (Table 2). It was a common Order of the litter invertebrate assemblage from tropical and subtropical regions, was rare an Order in temperate regions. In Wuzhishan Natural Reserve, litter thrips individuals accounting for 5.9% of the litter invertebrates, the relative abundance was maximum, only less than Acarina and Collembola.

**Table 2.** Species composition and density (Means ± SE) of litter-dwelling thrips in 6 natural reserves from different latitude (unit: individuals/m^2^).

Species	6	5	4	3	2	1*
*Acallurothrips* sp.	0.0a	0.0a	0.0a	0.0a	0.0a	0.3±0.3a**
*Adraneothrips russatus*	0.0b	0.0b	0.0b	0.0b	0.0b	1.0±0.7a
*Adraneothrips chinensis*	0.0a	0.0a	0.0a	3.3±3.3a	2.3±1.2a	3.5±1.5a
*Apelaunothrips lieni*	0.0d	0.0d	5.5±2.1bc	7.8±7.1ab	19.3±9.5a	1.0±0.5c
*Baenothrips asper*	0.0a	0.0a	0.0a	0.0a	0.0a	0.5±0.5a
*Heliothripoides reticulates*	0.0a	0.0a	0.0a	0.0a	0.0a	0.3±0.3a
*Holothrips* sp.	0.0a	0.0a	0.0a	0.3±0.3a	0.0a	0.0a
*Holurothris morikawai*	0.0b	0.0b	0.0b	0.0b	3.0±2.7a	0.0b
*Hoplothrips* sp.	0.0d	0.5±0.3bc	2.3±2.0ab	5.5±2.4a	0.0d	1.8±1.5ab
*Hyidiothrips japonicus*	0.0b	0.0b	0.0b	0.0b	0.0b	7.5±3.3a
*Karnyothrips flavipes*	0.0a	0.0a	0.0a	0.5±0.3a	1.0±1.0a	0.3±0.3a
*Psalidothrips ascitus*	0.0c	0.0c	42.5±26.9a	0.0c	12.3±5.4b	21.3±18.2ab
*Psalidothrips simplus*	0.0a	0.0a	0.0a	0.3±0.3a	5.0±5.0a	0.0a
*Psalidothrips* sp.	0.0a	0.0a	0.0a	0.0a	0.3±0.3a	0.3±0.3a
*Preeriella parvula*	0.0b	2.5±2.2ab	0.0b	71.5±71.2a	0.5±0.5ab	0.0b
*Stephanothrips japonicus*	0.0c	0.0c	4.3±3.3b	22.3±8.1a	2.5±1.1b	1.0±0.6b
*Terthrothrips palmatus*	0.0b	0.0b	0.0b	0.0b	0.0b	2.0±1.2a
*Mystrothrips flavidus*	0.0b	0.0b	0.0b	0.0b	1.3±1.3ab	1.8±0.9a
*Thrips* sp.	0.0b	0.0b	0.0b	1.8±0.3a	0.0b	1.6±0.8a
Thrips larvae	0.0c	0.5±0.3c	37±12.3ab	14.8±10.5b	31.8±11.7ab	49.3±7.3a

*1. Wuzhishan Natural Reserve, Hainan Province; 2. Nankunshan Natural Reserve, Guangdong Province; 3. Shennongjia Natural Reserve, Hubei Province; 4. Jigongshan Natural Reserve, Henan Province; 5. Yunmengshan National Forest Park, Beijing; 6. Changbaishan Natural Reserve, Jilin Province.

**Values in a row followed by the same letters indicate no significant difference at 0.05 level of probability (ANOVA, Tukey HSD) and values with standard errors.

In total, 19 species of litter-dwelling thrips (1578 individuals), representing 16 genera and 2 families, were collected during the survey period (Table 2). Most species and genera belonged to the family Phlaeothripidae (18 and 15 respectively); these phlaeothripids live as fungus-feeders in leaf litter. Of the family Thripidae 22 individuals were collected; these thripids are flower-living or leaf-feeding and only enter the litter or soil to pupate.

Species composition and density of fungus-feeding thrips were different in the six natural reserves (Table 2). In Wuzhishan Natural Reserve, 371 individuals of 14 species and 12 genera were collected in leaf litter. Among them, *Psalidothrips ascitus* and *Hyidiothrips japonicus* were dominant species, accounting for 48.6% and 17.1% of the total adult thrips individuals, respectively. *Heliothripoides reticulates* and *Terthrothrips palmatus* were collected only in this reserve. In three natural reserves located in subtropics, 10 genera and 12 species were collected: *Preeriella parvula*, *Psalidothrips ascitus*, *Apelaunothrips lieni*, *Stephanothrips japonicus* were dominant species, accounting for 33.5%, 19.9%, 15.1% and 13.5% of the total adult thrips individuals, respectively. In Yunmengshan, 14 individuals in 2 species, *Preeriella parvula* and *Holothrips* sp., were collected. No leaf-litter thrips were collected in Changbaishan Natural Reserve, the most northern of the six sites.

### Diversity indices of litter-dwelling thrips

According to species number and individuals of each species, Shannon-Wiener diversity index, Simpson dominance index and Pielou evenness index were applied to analyze community structure of fungus-feeding thrips (Table 3). The order on the average species numbers per plot of fungus-feeding thrips was tropics > subtropics > temperate (n=25, *p*<0.05). Mean density of fungus-feeders per plot in tropics and subtropics was significantly higher than in temperate region (n=25, *p*<0.05), but the average density was not significantly different between tropical and subtropical zones (n=25, *p*>0.05). The diversity of fungus-feeding thrips in the tropics and subtropics was much higher than in the temperate area, based on the comparsions of Shannon-Wiener diversity index (*H*’), Pielou eveness index (*J*), and Simpson dominance index (*D*).

**Table 3.** The comparison of diversity indices of fungus-feeding thrips in five natural reserves from different latitude.

Diversity indices	6	5	4	3	2	1*
number of species/plot	0	1.2±0.4 d	3.4±0.5 c	5.4±0.4 b	5.8±0.2 b	8±0.9 a**
Density (mean±SE)	0	3.6±2.4 b	91.6±37.3 a	127.9±59.9 a	78.9±30.3 a	93.0±22.4 a
No. of species	0	2	4	8	10	14
Shannon-Wiener Diversity index *H*’	-	1.149	1.6207	1.995	2.424	2.202
Simpson's dominant index *D*	-	0.476	0.6152	0.638	0.7492	0.659
Pielou's equality index *J*	-	0.725	0.698	0.601	0.7008	0.564

*1. Wuzhishan Natural Reserve, Hainan Province; 2. Nankunshan Natural Reserve, Guangdong Province; 3. Shennongjia Natural Reserve, Hubei Province; 4. Jigongshan Natural Reserve, Henan Province; 5. Yunmengshan National Forest Park, Beijing; 6. Changbaishan Natural Reserve, Jilin Province.

**Values in a row followed by the same letters indicate no significant difference at 0.05 level of probability (ANOVA, Tukey HSD) and values with standard errors.

## Discussion

Extant insects of the order Thysanoptera include approximately 6000 described species worldwide, classified into nine families (ThripsWiki 2014). At least 2500 species are placed in the families Phlaeothripidae and Merothripidae, and many of these are found in forest litter where they feed on fungi (Mound 2005). The systematics of litter-dwelling thrips has been well studied by Mound and Palmer (1983), Mound (2013) and Mound and O'Neill (1974). These litter thrips are especially diverse in some areas of the tropics and subtropics, which were proved by some qualitatively extensive surveys with the purpose of extracting abundant specimens to describe new taxa and study morphological variation. For example, almost 50 species in eight genera of litter thrips were described from a single area of forest 50 km in diameter in southern Brazil (Mound 1977). Two merothripid species and 15 phlaeothripid species were recorded from Jinmuji forest, Kanagawa Prefecture, Japan (Okajima and Urushihara 1992). Fifty thrips species, including twenty-six undescribed species, were recovered from various forest types and microhabitats in a single locality of subtropical eastern Australia (Tree and Walter 2012).

In recent years, we conducted a series of investigations by quantitative sampling methods to survey the species diversity of leaf-litter thrips in China. We found these litter-dwelling thrips to be a common group of litter macro-invertebrates, with high species diversity and relative abundance in Guangdong Province of subtropical China. For example, the numbers of these thrips accounted for 3% to 13.5% of total litter macroinvertebrate individuals caught in four different forest types of two natural reserves (Li et al. 2004a, b). In another natural forest, the relative abundance of Thysanoptera was 5.0%, representing 10 genera and 12 species (Wang et al. 2008). In an urban forest remnant, 25 species of 19 genera of leaf-litter thrips were collected, and these constituted 6.5% of total litter macro-invertebrate individuals caught (original totals 1413 thrips out of 21817 individual macro-invertebrates) (Wang et al. 2007; Wang and Tong 2012). In the present work, the relative abundance of Thysanoptera was over 3.0% in four natural reserves from the subtropical and tropical zones, and reached 5.9% in the tropical zone of Wuzhishan Natural Reserve (Hainan Province), only less than Acarina and Collembola. In contrast, it was only 0.3% in the warm temperate zone of Yunmengshan National Forest Park (Beijing) and no individual was collected in mid temperate zone of Changbaishan Natural Reserve (Jilin Province). 14 species and 12 genera of fungus-feeding thrips were collected from leaf litter of tropical China, 10 genera and 12 species in the subtropics, but only 2 species in the warm temperate zone. No species was collected in mid temperate zone. The results indicate that species number and relative abundance increase with decreasing latitude.

In the New World, the fauna of litter thrips is represented primarily by three genera, Eurythrips, Terthrothrips and Tylothrips (Mound 1976, 1977). In contrast, samples taken in the Old World rarely include these genera, whereas species of Apelaunothrips and Adraneothrips are often abundant, with species of Psalidothrips and Zemiathrips found commonly in the litter of sclerophyll forests in Australia (Mound 2002; Tree and Walter 2012). A total of 128 species in 43 genera belonging to 2 families of litter thrips were documented from China. Fauna and Zoogeographical analyses indicated that fungus-feeding thrips are diverse in China. *Psalidothrips* and *Apelaunothrips* are the dominant genera, with 9 species and 8 species, respectively. Relative abundance also can provide assemblage composition and fauna characteristics, and refers to the total number of specimens for a particular species divided by the total number of all litter thrips. *Psalidothrips ascitus* and *Hyidiothrips japonicus* were dominant species in samples from the tropics, accounting for 48.6% and 17.1% of the total adult thrips individuals, respectively *Heliothripoides reticulates* and *Terthrothrips palmatus* were collected only in this reserve. In subtropics, *Preeriella parvula*, *Psalidothrips ascitus*, *Apelaunothrips lieni*, *Stephanothrips japonicus* were dominant species, accounting for 33.5%, 19.9%, 15.1% and 13.5% of the total adult thrips individuals, respectively. In the warm temperate zone, a few individuals were collected of *Preeriella parvula* and *Holothrips*.

A total of five distribution patterns can be recognized among the 15 genera of litter-dwelling thrips in six nature reserves. Genera with a Pan-tropical distribution are most abundant, including the following: *Acallurothrips*, *Adraneothrips*, *Baenothrips*, *Holothrips*, *Karnyothrips*, *Preeriella*, *Stephanothrips* and *Terthrothrips*. These genera are found in the tropical and subtropical areas of Asia-Africa-America. *Apelaunothrips* is distributed in the tropical and subtropical areas of Asia-Africa. *Hyidiothrips* and *Psalidothrips* occur in the warmer areas of Asia and America. *Heliothripoides*, *Holurothrips* and *Mystrothrips* are distributed across tropical or subtropical Asia, being found in China, Japan, Korea and the India-Malaya area. Excluding the genera with cosmopolitan distributions (*Hoplothrips*), almost all litter-dwelling genera are found in tropical and subtropical areas. Differing from geographic distribution patterns at genus level, the 18 species of fungus-feeding thrips were divided into four distribution patterns, according to species currently distribution areas. Endemic to South China distribution: *Apelaunothrips lieni*, *Adraneothrips chinensis* and *Terthrothrips palmatus*. Eastern Asia distribution: *Adraneothrips russatus*, *Holurothrips morikawai*, *Hyidiothrips japonicus*, *Psalidothrips simplus*, *Stephanothrips japonicus* and *Mystrothrips flavidus* recorded in Japan and South China. Tropical Asia distribution: *Heliothripoides reticulates*, *Psalidothrips ascitus* and *Preeriella parvula* recorded in India-Malaya area or Japan. Tropical Asia, Africa and America disconnected distribution: *Baenothrips asper* and *Karnyothrips flavipes*. These zoogeographic analyses indicate that litter-dwelling thrips possess tropical and subtropical characteristics. Litter-dwelling thrips inhabiting forest litter usually have weak flight ability, some species are even wingless, but individual genera or even species are sometimes found in two disconnected continents. The causes have been discussed by Wang and Tong (2012).

The species richness of litter-dwelling thrips in our quantitative investigation at six locations across a broad latitudinal gradient reveal an increase with decreasing latitude, as reported for many other taxonomic groups of invertebrates, prominent examples include termites (Collins 1989), butterflies (Sime and Brower 1998) and springtails (Sun et al. 2013). However, we also found that the density of litter-dwelling thrips peaked at our mid-latitude sites (Shennongjia Natural Reserve), with no individual collected at the northern extreme. Individuals of oribatid mites, springtails and soil nematodes were richest in the warm temperate regions, and they could be found from polar to equatorial regions (Bardgett and Wardle 2010); a similar conclusion was made concerning leaf-associated aquatic hyphomycetes (Jabiol et al. 2013). The mechanisms underlying such patterns are still not fully understood, but several explanations have been proposed, such as habitat relationship, body size of organisms, solar energy, temperature and precipitation, etc (Turner 2004; Willig et al. 2003). We think that temperature might be a limiting factor for the distribution of litter-dwelling thrips in the northern temperate zone, particularly areas with snow cover in winter. In tropical regions, particularly complicated climatic conditions lead to dramatic fluctuation among different seasons (Wang and Tong 2012), and this could decrease number of individuals of litter thrips.
